# A Paper Man

**DOI:** 10.1007/s10912-019-09552-2

**Published:** 2019-02-26

**Authors:** Yusuf Patrick

**Affiliations:** Imperial College School of Medicine, Imperial College London, South Kensington Campus, Sir Alexander Fleming Building, London, SW7 2DD UK

They’ve written books about me

Binary tomes that fill servers across the country

Each episode written by a stranger

Telling you who I am

It doesn’t matter you’ll never read them

Just the headlines for today

Alcoholic, Leg ulcer, GERD, PD

So many labels that you’ve lost sight of me

You sit in front of me and say “this is the first time we’ve met”

That’s a lie that you tell yourself

Already I’m in a box with the rest of them

A proforma waiting for the blanks to be colored in

But I’ve met you before as well

The same mask of soothing smile and attentive eye

Wanting me to unpack my trauma

Before giving me green slips to redirect my baggage

The next question is about my father

They tell me that’s why I have issues with control

Thirty years of my life defined by a man I can’t remember

A life reduced to one word “abuse”

He’s described as my Frankenstein

But he was a Mariner passing on his albatross

Now I carry it fearing who will inherit it next

A modern day Sisyphus

But that’s not what you expect

You expect the coarseness and the rough

To fit with your stereotype of who I am

And what kind of patient would disappoint

Now you give me advice

Exerting control on the chaos of my life

No medicine to turn back time

Or stop the march of entropy

Finished with me you turn away

Putting the final touches to my biography

Ushering me out the door with a smile

You hope this is your last chapter in my story

A world blind to the books on every corner

A sea of faces that make me feel more alone

A life resolved in a line of black and white

A footnote in another forgotten story

